# *Histoplasma capsulatum* in Bat Species in Portugal

**DOI:** 10.3390/vetsci12020094

**Published:** 2025-01-25

**Authors:** Jaqueline T. Bento, Ana Cláudia Coelho, Hugo Rebelo, João R. Mesquita

**Affiliations:** 1 School of Medicine and Biomedical Sciences (ICBAS), University of Porto, 4050-313 Porto, Portugal; jtbento@icbas.up.pt; 2 Department of Veterinary Sciences, University of Trás-Os-Montes e Alto Douro (UTAD), Quinta dos Prados, 5000-801 Vila Real, Portugal; accoelho@utad.pt; 3 Animal and Veterinary Research Centre (CECAV), Quinta dos Prados, 5000-801 Vila Real, Portugal; 4 Associate Laboratory for Animal and Veterinary Sciences (AL4AnimalS), 1300-477 Lisboa, Portugal; 5 BIOPOLIS Program in Genomics, Biodiversity and Land Planning, Research Center in Biodiversity and Genetic Resources (CIBIO), Campus de Vairão, 4485-661 Vairão, Portugal; herebelo@ciencias.ulisboa.pt; 6 CE3C—Centre for Ecology, Evolution and Environmental Changes & CHANGE—Global Change and Sustainability Institute, Departamento de Biologia Animal, Faculdade de Ciências, Universidade de Lisboa, 1749-016 Lisboa, Portugal; 7 Centro de Estudos de Ciência Animal (CECA), Instituto de Ciências, Tecnologias e Agroambiente (ICETA), Universidade do Porto (UP), Rua D. Manuel II, Apartado 55142, 4051-401 Porto, Portugal

**Keywords:** *Histoplasma capsulatum*, guano, bats, Portugal

## Abstract

Histoplasmosis, caused by the fungus *Histoplasma capsulatum*, poses health risks to various mammals, including humans. Bats are primary wild carriers of *Histoplasma capsulatum*, playing a crucial role in its epidemiology. However, fecal shedding in Europe remains poorly studied, with no data available for Portugal. This study analyzed 285 guano samples from 22 bat species, collected across Portuguese regions between 2014 and 2018, using a nested PCR assay. Despite using a sensitive method, no positive samples were detected. These results align with other European studies, suggesting that *Histoplasma capsulatum* circulates at low levels in European bat populations. However, they contrast with findings from regions like Brazil and Mexico, where the fungus is more prevalent due to differing geographic, climatic, and ecological factors. The absence of *Histoplasma capsulatum* in Portuguese bat guano highlights the importance of local environmental conditions and raises questions about its distribution in Europe. Although bats can harbor zoonotic pathogens, our findings suggest they do not shed *Histoplasma capsulatum* in Portugal. Continuous monitoring and research are essential in understanding infectious disease dynamics. Targeted surveillance in caves could improve early detection and management strategies for potential histoplasmosis outbreaks, contributing to public health efforts in these ecosystems.

## Simple Summary

Histoplasmosis, caused by the fungus *Histoplasma capsulatum*, can impact animals and humans, with bats as known carriers. We tested 285 bat guano samples from Portugal and found no positive cases, consistent with low detection rates in Europe. This suggests that bats in Portugal are unlikely to spread *Histoplasma capsulatum* and highlights the need for continued monitoring.

## 1. Introduction

Histoplasmosis is a systemic infection found in numerous locations worldwide [1,2], caused by the dimorphic ascomycete fungus *Histoplasma capsulatum*, belonging to the family Ajellomycetaceae and the order Onygenales [3,4]. This fungal pathogen dimorphism is induced by temperature variations during its growth, involving a transition between a filamentous phase and a yeast phase [3]. This shift involves changes in cellular morphology and cell wall composition, which are critical factors in the virulence of fungi such as *H. capsulatum* [5]. In its environmental, dimorphic mold phase, *H. capsulatum* produces microconidia, the latter being smooth-walled and 2 to 4 μm in diameter and serving as the infectious elements acquired via inhalation. The yeast phase, representing the parasitic form, consists of oval budding cells measuring 2 to 4 μm, predominantly observed within macrophages and histiocytes, indicative of its capacity for persistent latency [6].

*Histoplasma capsulatum* was traditionally classified into three varieties: *H. capsulatum* var. *capsulatum*, *H. capsulatum* var. *duboisii*, and *H. capsulatum* var. *farciminosum* [1,3,7]. However, recent studies have revealed extensive genetic diversity within the *capsulatum* variety, suggesting the existence of several new species. Using whole-genome sequencing, *H. capsulatum* var. *capsulatum* has been reclassified into four distinct species: *H. capsulatum* sensu stricto (known as the Panama or H81 lineage), *H. mississippiense* (NAm1 lineage), *H. ohiense* (NAm2 lineage), and *H. suramericanum* (LAmA lineage) [8,9].

The morphologic transition of *H. capsulatum* from mold to yeast is essential to its virulence. Experimental studies have shown that this conversion to the yeast form is crucial in establishing disease [4,10]. This conversion alters the composition of the cell wall, increasing α-(1,3)-glucan, associated with the fungus’s virulence. Studies have shown that the α-(1,3)-glucan synthase gene (AGS1) is responsible for producing this polymer, which is essential in the pathogenicity of *H. capsulatum* [4,11]. When this gene is disrupted or its expression is silenced, the growth of the yeast in macrophages in vitro is impaired, as well as its ability to colonize the lungs of rodents. Additionally, *H. capsulatum* expresses a calcium-binding protein (CBP1) for growth in macrophages and survival in the host. The fungus also produces the protein Yps3, which aids in its virulence. The α-(1,3)-glucan on the yeast surface helps the fungus evade detection by the host’s immune system, contributing to its pathogenicity [4,12,13].

In immunocompetent individuals, the infection often remains asymptomatic, with fatal outcomes being uncommon [14]. However, in immunocompromised individuals, such as solid organ transplant recipients or those taking immunosuppressive medications, the infection can become severe and spread, raising the risk of serious complications [15]. People with HIV/AIDS are especially vulnerable; in the United States, between 2002 and 2017, the mortality rate for HIV-positive individuals was approximately 37% [14]. The clinical manifestations includes acute pulmonary histoplasmosis, chronic cavitary pulmonary histoplasmosis, granulomatous mediastinitis, mediastinal fibrosis, and even pericarditis in some cases [16,17]. The symptoms include fever, headache, weakness, substernal chest discomfort, and dry cough [17].

Histoplasmosis is predominantly found in the Americas, Africa, and Asia. Although histoplasmosis is not endemic in Europe, it is typically considered an imported disease, and there have been reports of small foci and potential autochthonous human cases in countries such as Italy [1,3], the United Kingdom, Germany, Turkey, Switzerland, Spain, and France [1,15,18,19,20], with the vast majority of cases detected in Spain, France, and Italy. A cat in Europe presented with systemic signs, including an abdominal mass and granulomatous inflammation, and was diagnosed post mortem with histoplasmosis, marking the first reported case in felines within Europe [21].

The disease is prevalent in the Mississippi and Ohio valleys in the United States, as well as in Central and South America, India, Southeast Asia, and parts of Africa. Histoplasmosis is acquired through the inhalation of microconidia usually found in soils, bird feces, and bat guano and affects various mammalian hosts, including dogs, rodents, cats, horses, marsupials, and bats [3,22,23].

Bats are considered the primary wild mammalian carriers of *H. capsulatum* [3], playing a significant role in the epidemiology of histoplasmosis. These animals become infected by inhaling fungal spores, which can disseminate throughout their bodies, including the intestines, and are subsequently released into the environment via their feces. Their nitrogen-rich guano not only facilitates further fungal growth but also enables the infection of new hosts, underscoring the crucial role that bats play in the epidemiology of histoplasmosis [24,25]. Bats harbor several pathogens in their feces, serving as significant sources of environmental contamination [26]. Studies have shown that randomly captured bats can develop disseminated histoplasmosis, as evidenced by positive cultures from key organs of their immune system, such as the liver and spleen [16]. As known reservoirs for *H. capsulatum*, bats not only promote fungal growth in caves through their waste but also introduce infectious spores into guano-covered cave surfaces. Bats can carry and spread *H. capsulatum* even outside of caves [27]. The infective microconidia of *H. capsulatum* become aerosolized when contaminated soil is disturbed, and once they have been inhaled and have reached the alveoli in the lungs, they transform into budding yeast forms [28]. Unlike birds, bats can both acquire and transmit the fungus, aiding its incorporation into new ecological niches in favorable environments [29]. Numerous reports highlight histoplasmosis outbreaks among individuals exposed to accumulated bat feces, particularly among workers involved in cleaning or demolishing buildings and tourists visiting caves with large bat colonies [30,31,32,33,34].

Only a few studies have assessed the role of European populations of bats as reservoirs for *H. capsulatum* [35,36], and to the best of our knowledge no study has ever been conducted in the Iberian bat species. Given the important role that bats play in the epidemiology of histoplasmosis in animals and humans, this study aimed to investigate the presence of *H. capsulatum* in guano samples from different bat species in Portugal.

## 2. Materials and Methods

### 2.1. Study Population

**Figure 1:**
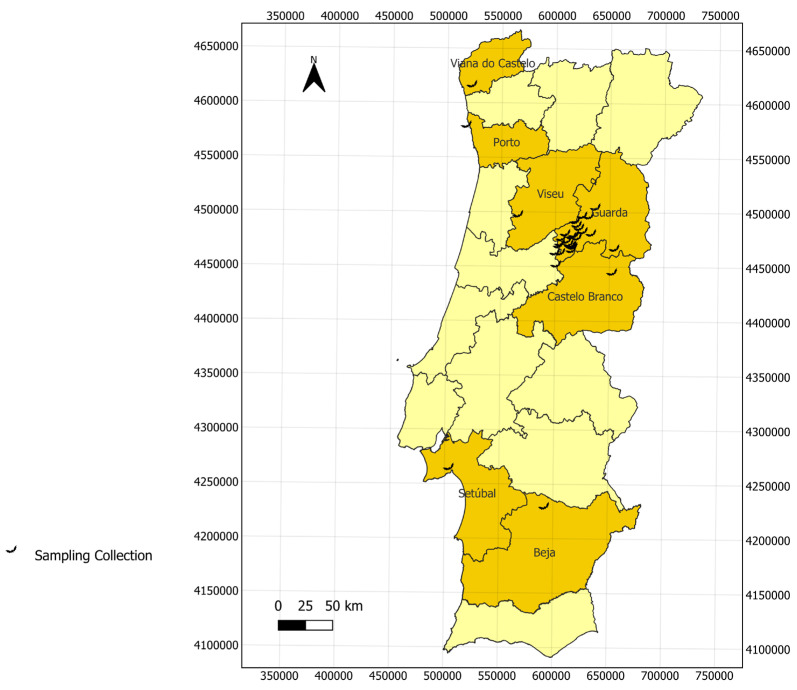
Geographical distribution of guano samples collected in Portugal. Dark yellow represents the districts were the samples were collected from and light yellow the remaining districts of mainland Portugal.

A study was carried out involving 285 guano samples collected between 2014 and 2018 in 33 different locations in the Viana do Castelo (*n*= 2), Porto (*n* = 4), Viseu (*n* = 22), Guarda (*n* = 174), Castelo Branco (*n* = 77), Setúbal (*n* = 3), and Beja (*n* = 3) regions of Portugal (Figure 1). Bats were captured using mist nets or harp traps at roost exits. Individual bats were placed in clean cotton bags, from which guano pellets were gathered. These pellets were stored in 2 mL tubes with silica beads and kept at −20 °C until further analysis [37]. Bat species were visually identified by two experts on the team. Additionally, to confirm species designation, a 3 mm wing punch was non-lethally taken from five individuals from each species that had first been visually identified. Wing punches were stored in 96% ethanol and used for DNA extraction, followed by the amplification and sequencing of two COI gene fragments [38].

All actions related to the capture and handling of bats were carried out in full compliance with the permits issued by the Instituto da Conservação da Natureza e das Florestas, ensuring strict adherence to the regulations and guidelines established by the conservation authority (license number: l 274/2023/CAPT).

### 2.2. DNA Extraction

Genomic DNA was extracted using a custom protocol. This protocol began with incubation in a lysis buffer (0.1 M Tris–HCl, 0.1 M EDTA, 0.01 M NaCl, 1% N-lauroylsarcosine, pH 7.5–8), followed by the removal of inhibitors using Inhibitex tablets (Qiagen, Hilden, Germany), cell lysis, DNA precipitation, and washing, which were performed with the E.Z.N.A. Tissue Kits (Omega Bio-Tek, Norcross, GA, USA). The extraction protocol was started by adding one fecal pellet to 800 µL of lysis buffer. The samples were homogenized, vortexed, and incubated at 70 °C for 30 min in a dry bath. Thereafter, samples underwent a short spin, and up to 700 µL of supernatant was transferred to a new tube with a quarter of an Inhibitex tablet. The mixture was vortexed for 1 min and centrifuged again at 12,000× *g* for 30 s. Up to 500 µL of the supernatant was then transferred to a new tube, and 25 µL of OB Protease was added. The subsequent steps followed the kit instructions, except for DNA being eluted twice into two separate tubes, each eluted in a 50 µL volume. DNA was extracted in batches of 23 samples, with one negative control, where no fecal pellet was used. The extracted DNA was placed in 96-well plates.

### 2.3. Molecular Detection of H. capsulatum

To detect *H. capsulatum*, we employed a nested PCR with the outer primer set HcI (5′-GCG TTC CGA GCC TTC CAC CTC AAC-3′) and HcII (5′-ATG TCC CAT CGG GCG CCG TGT AGT-3′) and the inner primers HcIII (5- GAGATCTAGTCGCGGCCA GGT TCA-3′) and HcIV (5′-AGG AGA GAA CTG TAT CGG TGG CTT G-3′), which amplified a 210 bp final gene PCR product [39]. PCR reactions were performed on a thermocycler (Bio-Rad, Hercules, CA, USA). Reactions mixtures were prepared using SpeedySupreme NZYTaq 2x Green Master Mix (NZYTech, Lisbon, Portugal) according to the manufacturer’s instructions, with the appropriate primer sets for each round. The first-round cycling conditions included an initial denaturation at 95 °C for 5 min, followed by 40 cycles at 94 °C for 2 s, 50 °C for 5 s, and 72 °C for 5 s, ending with a final extension at 72 °C for 10 min. The second round follow the same conditions, except for the annealing temperature that was changed to 65 °C.

Following PCR amplification, DNA fragments were separated by electrophoresis on 1.5% agarose gels stained with Xpert Green Safe DNA gel dye (GRiSP^®^, Porto, Portugal). The electrophoresis was run at a constant voltage of 120 V for 25 min. The results were visualized by exposing the gels to UV light.

For the positive control, a synthetic oligonucleotide corresponding to the target sequence of *H. capsulatum* was used, covering the region amplified by the external/internal primers HcIII and HcIV, resulting in a 210 bp product.

### 2.4. Sequencing Analysis

Amplicons of the expected size were purified using the GRS PCR & Gel Band Purification Kit (GRiSP^®^, Porto, Portugal). After purification, bidirectional sequencing was performed with the Sanger method using the appropriate internal primers for the target gene. Sequences were then aligned with the help of the BioEdit Sequence Alignment Editor v7.1.9 software package, version 2.1v, and compared with those in the NCBI (GenBank) nucleotide database (https://blast.ncbi.nlm.nih.gov/Blast.cgi, accessed on 10 September 2024).

## 3. Results

In this study, a total of 285 stool samples were collected between 2024 and 2018 from bats in seven regions of Portugal. From the visual identification followed by COI DNA barcode analysis, it was determined that bats belonged to 22 species from four families (Vespertilionidae, Rhinolophidae, Molossidae, and Miniopteridae) (Table 1).

In the screening of the total 285 guano samples for *H. capsulatum* by nested PCR, none were shown to be positive for *H. capsulatum*.

## 4. Discussion

This study investigated the presence of *H. capsulatum* in 285 guano samples collected from 22 bat species across seven districts in Portugal, with no positive cases detected using nested PCR. The results presented here should be interpreted with caution. Although we used a highly sensitive and specific approach to *H. capsulatum* detection, this pathogen could be present in quantities below the method’s detection limit. The detection method could also be a factor in the negative results; the definitive method for detection histoplasmosis involves conventional techniques such as fungal culture and histopathological analysis. Although culturing *H. capsulatum* is highly specific and reliable, it requires advanced biosafety level 3 facilities, highlighting the need for alternative or complementary diagnostic approaches that provide quicker results and are less dependent on specialized infrastructure [40].

These results align with the limited literature available in databases on *H. capsulatum* in Europe. For instance, a study conducted on bat samples in France reported that only 1 out of 83 samples tested positive for *H. capsulatum* [35], highlighting the rarity of detection in European bat populations.

On the other hand, the results stand in contrast to findings from other studies on bat samples from other non-European regions, such as Brazil [3,16,25,32] and Mexico [24,41]. This divergence could be due to several factors, including geographic differences in fungal distribution or climatic and ecological conditions in each region that influence the prevalence of the fungus. While Portugal has a more Mediterranean climate (Köppen climate classification Csa/Csb) that limits the prevalence of *H. capsulatum*, Brazil, with its humid tropical climate (Köppen: Af/Aw/Cfa/BSh), provides ideal conditions for fungal development. Mexico, with its climatic diversity (Köppen: Af/Csa/BSh/Bw), has regions where conditions are favorable for *H. capsulatum*, especially the tropical areas in the south; however, the arid zones of the north are less favorable. This comparison highlights how climatic conditions influence the epidemiology of histoplasmosis in different regions.

Although few studies have been conducted in bat reservoirs in Europe, studies performed in humans have identified potential autochthonous cases in countries such as Italy, the United Kingdom, Germany, Turkey, Switzerland, Spain, and France [1,15,18,19,20], of which 80.7% of the cases were diagnosed in Spain (49.1%), France (19.3%), and Italy (12.3%) [15]. The results from this previous studies, including case reports from Portugal, have demonstrated the presence of *H. capsulatum* in this geographical area [1,15,18,20,42]. However, most cases reported in Portugal involved individuals who had lived for extended periods in Africa, where *H. capsulatum* var. duboisii, the causative agent of African histoplasmosis, is more commonly found. *H. capsulatum* var. duboisii is not native to Europe, and cases in Europe, particularly in Portugal, are often linked to travel or residency in Africa [42,43]. The presence of this variant further underscores the influence of geography on the distribution of *H. capsulatum* and the epidemiology of histoplasmosis.

Although this study shows no evidence of the shedding of *H. capsulatum* in the stools of bats in Portugal, the pathogen’s presence in very low concentrations, potentially below the detection threshold, cannot be ruled out, further emphasizing the need for continued investigations with alternative methods or more sensitive approaches, to detect *H. capsulatum* in low-prevalence areas.

## 5. Conclusions

In conclusion, despite the extensive testing of guano samples in Portugal, no positive results for *H. capsulatum* were found. This absence raises important questions regarding the distribution of this pathogen within the region. Given the ecological conditions of caves and the potential habitats for this fungus, it is crucial that we consider the implications for human health and the possibility of zoonotic transmission. Although our study indicates a lack of *H. capsulatum* presence in these specific samples, further research is necessary to better understand the epidemiology of this pathogen in Portugal. Implementing monitoring programs in cave ecosystems could be beneficial in the early detection and management of future outbreaks.

## Figures and Tables

**Table 1:** Description, number, and percentages of bat species included in the study with indication of the main roost type used by each species.

Species	Family	Number	Percentage	Roost Type
*Rhinolophus euryale*	Rhinolophidae	8	2.8	Cave-dwelling
*Rhinolophus ferrumequinum*	Rhinolophidae	16	5.6	Cave-dwelling
*Rhinolophus hipposideros*	Rhinolophidae	4	1.4	Cave-dwelling
*Barbastella barbastellus*	Vespertilionidae	14	4.9	Tree-dwelling
*Eptesicus isabellinus*	Vespertilionidae	3	1.1	Several
*Hypsugo savii*	Vespertilionidae	17	6.0	Rock-dwelling
*Eptesicus serotinus*	Vespertilionidae	13	4.6	Several
*Myotis blythii*	Vespertilionidae	2	0.7	Cave-dwelling
*Myotis daubentonii*	Vespertilionidae	17	6.0	Tree-dwelling
*Myotis emarginatus*	Vespertilionidae	13	4.6	Tree-dwelling
*Myotis escalerai*	Vespertilionidae	24	8.4	Tree-dwelling
*Myotis myotis*	Vespertilionidae	8	2.8	Cave-dwelling
*Myotis mystacinus*	Vespertilionidae	1	0.4	Tree-dwelling
*Nyctalus lasiopterus*	Vespertilionidae	4	1.4	Tree-dwelling
*Nyctalus leisleri*	Vespertilionidae	24	8.4	Tree-dwelling
*Pipistrellus kuhlii*	Vespertilionidae	5	1.8	Several
*Pipistrellus pipistrellus*	Vespertilionidae	54	18.9	Several
*Pipistrellus pygmaeus*	Vespertilionidae	1	0.4	Several
*Plecotus auritus*	Vespertilionidae	27	9.5	Several
*Plecotus austriacus*	Vespertilionidae	17	6.0	Several
*Tadarida teniotis*	Molossidae	11	3.9	Rock-dwelling
*Miniopterus schreibersii*	Miniopteridae	2	0.7	Cave-dwelling
Total	N.A.	285	100	N.A.

N.A.: Not applicable.

## Data Availability

The data that support the findings of this study are available from the corresponding author upon reasonable request.

## References

[1] Wilmes D., Mayer U., Wohlsein P., Suntz M., Gerkrath J., Schulze C., Holst I., von Bomhard W., Rickerts V. (2022). Animal Histoplasmosis in Europe: Review of the Literature and Molecular Typing of the Etiological Agents. J. Fungi.

[2] Bahr N.C., Antinori S., Wheat L.J., Sarosi G.A. (2015). Histoplasmosis Infections Worldwide: Thinking Outside of the Ohio River Valley. Curr. Trop. Med. Rep..

[3] da Silva J.A., Scofield A., Barros F.D.N., de Farias D.M., Riet-Correa G., Bezerra Júnior P.S., Santos T.F.S., Tavares G.S.F., Trevelin L.C., da Paz G.S. (2021). Molecular Detection of *Histoplasma Capsulatum* in Bats of the Amazon Biome in Pará State, Brazil. Transbound. Emerg. Dis..

[4] Klein B.S., Tebbets B. (2007). Dimorphism and Virulence in Fungi. Curr. Opin. Microbiol..

[5] Oladele R.O., Ayanlowo O.O., Richardson M.D., Denning D.W. (2018). Histoplasmosis in Africa: An Emerging or a Neglected Disease?. PLoS Negl. Trop. Dis..

[6] Jeannette G., Brandt M.E. (2011). Histopathologic Diagnosis of Fungal Infections in the 21st Century. Clin. Microbiol. Rev..

[7] Weeks R.J., Padhye A.A., Ajello L. (1985). Histoplasma Capsulatum Variety Farciminosum: A New Combination for *Histoplasma Farciminosum*. Mycologia.

[8] Kidd S.E., Abdolrasouli A., Hagen F. (2023). Fungal Nomenclature: Managing Change Is the Name of the Game. Open Forum Infectious Diseases.

[9] Sepúlveda V.E., Márquez R., Turissini D.A., Goldman W.E., Matute D.R. (2017). Genome Sequences Reveal Cryptic Speciation in the Human Pathogen *Histoplasma Capsulatum*. MBio.

[10] Medoff G., Kobayashi G.S., Painter A., Travis S. (1987). Morphogenesis and Pathogenicity of *Histoplasma Capsulatum*. Infect. Immun..

[11] Hogan L.H., Klein B.S. (1994). Altered Expression of Surface Alpha-1,3-Glucan in Genetically Related Strains of Blastomyces Dermatitidis That Differ in Virulence. Infect. Immun..

[12] Brandhorst T.T., Wüthrich M., Finkel-Jimenez B., Warner T., Klein B.S. (2004). Exploiting Type 3 Complement Receptor for TNF-Alpha Suppression, Immune Evasion, and Progressive Pulmonary Fungal Infection. J. Immunol..

[13] Keath E.J., Painter A.A., Kobayashi G.S., Medoff G. (1989). Variable Expression of a Yeast-Phase-Specific Gene in *Histoplasma Capsulatum* Strains Differing in Thermotolerance and Virulence. Infect. Immun..

[14] Dao A., Kim H.Y., Halliday C.L., Oladele R., Rickerts V., Govender MMed N.P., Shin J.H., Heim J., Ford N.P., Nahrgang S.A. (2024). Histoplasmosis: A Systematic Review to Inform the World Health Organization of a Fungal Priority Pathogens List. Med. Mycol..

[15] Antinori S., Giacomelli A., Corbellino M., Torre A., Schiuma M., Casalini G., Parravicini C., Milazzo L., Gervasoni C., Ridolfo A.L. (2021). Histoplasmosis Diagnosed in Europe and Israel: A Case Report and Systematic Review of the Literature from 2005 to 2020. J. Fungi.

[16] Ruiz-Muñoz J.A., Rodríguez-Arellanes G., Ramírez J.A., Carreto-Binaghi L.E., Fusco-Almeida A.M., Mendes-Giannini M.J.S., García-Pérez B.E., Taylor M.L. (2024). Molecular Detection of *Histoplasma Capsulatum* in Organ Samples from Bats Randomly Captured in Urban Areas of Araraquara, São Paulo State, Brazil. Epidemiol. Infect..

[17] Kauffman C.A. (2007). Histoplasmosis: A Clinical and Laboratory Update. Clin. Microbiol. Rev..

[18] Alados J.C., Miranda C., Ortiz F., Cano R. (1993). Disseminated Histoplasmosis in an AIDS Patient in Spain. Eur. J. Clin. Microbiol. Infect. Dis..

[19] Confalonieri M., Gandola L., Aiolfi S., Parigi P., Mazzoni A. (1994). Histoplasmin Sensitivity among a Student Population in Crema, Po Valley, Italy. New Microbiol..

[20] Mantovani A. (1972). Histoplasmosis in Europe. Ann. Soc. Belg. Med. Trop. (1920).

[21] Mavropoulou A., Grandi G., Calvi L., Passeri B., Volta A., Kramer L.H., Quintavalla C. (2010). Disseminated Histoplasmosis in a Cat in Europe. J. Small Anim. Pract..

[22] Brilhante R.S.N., Fechine M.A.B., Mesquita J.R.L., Cordeiro R.A., Rocha M.F.G., Monteiro A.J., Lima R.A.C., Caetano É.P., Pereira J.F., Castelo-Branco D.S.C.M. (2012). Histoplasmosis in HIV-Positive Patients in Ceará, Brazil: Clinical-Laboratory Aspects and in Vitro Antifungal Susceptibility of *Histoplasma Capsulatum* Isolates. Trans. R. Soc. Trop. Med. Hyg..

[23] Nacher M., Adenis A., Mc Donald S., Do Socorro Mendonca Gomes M., Singh S., Lopes Lima I., Malcher Leite R., Hermelijn S., Wongsokarijo M., Van Eer M. (2013). Disseminated Histoplasmosis in HIV-Infected Patients in South America: A Neglected Killer Continues on Its Rampage. PLoS Negl. Trop. Dis..

[24] Vite-Garín T., Estrada-Bárcenas D.A., Gernandt D.S., Reyes-Montes M.D.R., Sahaza J.H., Canteros C.E., Ramírez J.A., Rodríguez-Arellanes G., Serra-Damasceno L., Zancopé-Oliveira R.M. (2021). *Histoplasma Capsulatum* Isolated from Tadarida Brasiliensis Bats Captured in Mexico Form a Sister Group to North American Class 2 Clade. J. Fungi.

[25] Dos Santos B., Langoni H., da Silva R.C., Menozzi B.D., Bosco S.D.M.G., Paiz L.M., Augusto L.C.R., Richini-Pereira V.B. (2018). Molecular Detection of *Histoplasma Capsulatum* in Insectivorous and Frugivorous Bats in Southeastern Brazil. Med. Mycol..

[26] Brilhante R.S.N., Maia-Júnior J.E., Oliveira J.S., Guedes G.M.M., Silva A.L., Moura F.B.P., Sales J.A., Castelo-Branco D.S.C.M., Sidrim J.J.C., Cordeiro R.A. (2016). Yeasts from the Microbiota of Bats: A Focus on the Identification and Antimicrobial Susceptibility of Cryptic Species of Candida. J. Med. Microbiol..

[27] Jülg B., Elias J., Zahn A., Köppen S., Becker-Gaab C., Bogner J.R. (2008). Bat-Associated Histoplasmosis Can Be Transmitted at Entrances of Bat Caves and Not Only inside the Caves. J. Travel Med..

[28] Diaz H. (2018). Environmental and Wilderness-Related Risk Factors for Histoplasmosis: More Than Bats in Caves. Wilderness Environ. Med..

[29] Gugnani H.C., Denning D.W. (2023). Infection of Bats with *Histoplasma* Species. Med. Mycol..

[30] Huhn G.D., Austin C., Carr M., Heyer D., Boudreau P., Gilbert G., Eimen T., Lindsley M.D., Cali S., Conover C.S. (2005). Two Outbreaks of Occupationally Acquired Histoplasmosis: More than Workers at Risk. Environ. Health Perspect..

[31] Oliveira F.D.M., Unis G., Severo L.C. (2006). An Outbreak of Histoplasmosis in the City of Blumenau, Santa Catarina. J. Bras. Pneumol..

[32] Dias M.A.G., Oliveira R.M.Z., Giudice M.C., Netto H.M., Jordão L.R., Grigorio I.M., Rosa A.R., Amorim J., Nosanchuk J.D., Travassos L.R. (2011). Isolation of *Histoplasma Capsulatum* from Bats in the Urban Area of São Paulo State, Brazil. Epidemiol. Infect..

[33] Santos L., Santos-Martínez G., Magaña-Ortíz J.E., Puente-Piñón S.L. (2013). Acute Histoplasmosis in Three Mexican Sewer Workers. Occup. Med..

[34] Negroni R., Duré R., Ortiz Nareto A., Arechavala A.I., Maiolo E.I., Santiso G.M., Iovannitti C., Ibarra-Camou B., Canteros C.E. (2010). Histoplasmosis outbreak in Morón, Buenos Aires Province, Argentina. Rev. Argent. Microbiol..

[35] González-González A.E., Ramírez J.A., Aliouat-Denis C.M., Demanche C., Aliouat E.M., Dei-Cas E., Chabé M., Taylor M.L. (2013). Molecular Detection of *Histoplasma Capsulatum* in the Lung of a Free-Ranging Common Noctule (NYC-Talus Noctula) from France Using the Hcp100 Gene. J. Zoo Wildl. Med..

[36] Beguin H., Larcher G., Nolard N., Chabasse D. (2005). Chrysosporium Chiropterorum Sp. Nov., Isolated in France, Resembling Chrysosporium State of Ajellomyces Capsulatus (*Histoplasma capsulatum*). Med. Mycol..

[37] Raposeira H., Horta P., Heleno R., Rebelo H. (2023). Changing with the Times: Seasonal Environmental Gradients Unveil Dynamic Bat Assemblages and Vulnerability. Ecol. Evol..

[38] Rebelo H., Ferreira S., Amorim F., Horta P., Raposeira H., Santos H., Beja P., Mata V.A. (2020). Hidden in Our Pockets: Building of a DNA Barcode Library Unveils the First Record of Myotis Alcathoe for Portugal. Biodivers. Data J..

[39] Bialek R., Feucht A., Aepinus C., Just-Nübling G., Robertson V.J., Knobloch J., Hohle R. (2002). Evaluation of Two Nested PCR Assays for Detection of *Histoplasma Capsulatum* DNA in Human Tissue. J. Clin. Microbiol..

[40] Tobón A.M., Gómez B.L. (2021). Pulmonary Histoplasmosis. Mycopathologia.

[41] Taylor M.L., Chávez-Tapia C.B., Vargas-Yañez R., Rodríguez-Arellanes G., Peña-Sandoval G.R., Toriello C., Pérez A., Reyes-Montes M.R. (1999). Environmental Conditions Favoring Bat Infection with *Histoplasma Capsulatum* in Mexican Shelters. Am. J. Trop. Med. Hyg..

[42] Marques N., Lebre A., Marques F., Julião M., Freitas L., Malcata L., Rabadão E., da Cunha J.S. (2013). Isolated Oral Histoplasmosis Presenting as Fever of Unknown Origin in a Portuguese Hemodialysis Patient. Mycopathologia.

[43] Cardoso L., Silva C., Marques N., Veríssimo C. (2017). Tonsillar Ulceration as Manifestation of Disseminated African Histoplasmosis in an Immunocompetent Portuguese Host. Med. Mycol. Case Rep..

